# Relationship Between Serum Levels of Pancreatic Exocrine Enzymes on Admission and Long-Term Clinical Outcomes in Patients with Acute Decompensated Heart Failure

**DOI:** 10.3390/jcm14051500

**Published:** 2025-02-24

**Authors:** Masaru Hiki, Takatoshi Kasai, Akihiro Sato, Sayaki Ishiwata, Shoichiro Yatsu, Jun Shitara, Hiroki Matsumoto, Megumi Shimizu, Azusa Murata, Takao Kato, Shoko Suda, Hiroshi Iwata, Hiroyuki Daida

**Affiliations:** 1 Department of Cardiovascular Biology and Medicine, Juntendo University School of Medicine, Tokyo 113-8421, Japan; ma-hiki@juntendo.ac.jp (M.H.); ak-sato@juntendo.ac.jp (A.S.); s-ishiwata@juntendo.ac.jp (S.I.); hmatsumo@juntendo.ac.jp (H.M.); shimizumegumi1986@gmail.com (M.S.); azmurata@juntendo.ac.jp (A.M.); tkatou@juntendo.ac.jp (T.K.); ssuda@juntendo.ac.jp (S.S.); h-iwata@juntendo.ac.jp (H.I.); daida@juntendo.ac.jp (H.D.); 2 Cardiovascular Respiratory Sleep Medicine, Juntendo University Graduate School of Medicine, Tokyo 113-8421, Japan; 3 Department of Cardiovascular Management and Remote Monitoring, Juntendo University Graduate School of Medicine, Tokyo 113-8421, Japan; 4 Sleep and Sleep Disordered Breathing Center, Juntendo University Hospital, Tokyo 113-8421, Japan; 5 Department of Cardiology, Juntendo University Shizuoka Hospital, Shizuoka 410-2211, Japan; syatsu@juntendo.ac.jp (S.Y.); jshitara@juntendo.ac.jp (J.S.)

**Keywords:** amylase, hospitalization, lipase, mortality

## Abstract

**Background/Objectives**: Heart failure (HF) can damage organs because of poor perfusion and/or congestion. The interactions between HF and other organs have recently been studied; however, data on the interaction between HF and pancreatic exocrine function, which may affect fat and protein absorption and malnutrition, are scarce. We previously showed that the serum levels of pancreatic exocrine enzymes, as suggestive of pancreatic exocrine function, were low and associated with malnutrition or congestion in hospitalized patients with acute decompensated HF (ADHF). This study investigated the relationship between the serum levels of pancreatic exocrine enzymes and long-term outcomes in patients with ADHF. **Methods**: We collected serum levels of pancreatic exocrine enzymes (amylase and lipase) from patients who were admitted to the cardiac intensive care unit due to ADHF. Patients undergoing dialysis and those with neoplasms were excluded. Patients were categorized as having high or low pancreatic exocrine enzyme levels in the first quartile upon admission. The association between low serum pancreatic exocrine enzyme levels at admission and the composite of death and ADHF readmission was assessed. **Results**: Of the 146 patients, 37 (25.3%) and 36 (24.7%) had low amylase and lipase levels, respectively. Patients with low lipase levels showed worse cumulative event-free survival than those with high lipase levels (*p* < 0.001). A low lipase level was associated with worse outcomes (hazard ratio: 1.96; *p* = 0.012). **Conclusions**: These findings suggest that low serum lipase levels may be a predictor of long-term outcomes in patients with ADHF.

## 1. Introduction

Despite advances in the pathophysiology of heart failure (HF) and an expanding array of evidence-based guideline-directed treatment options, once patients develop HF, their rates of hospitalization and mortality remain relatively high [1]. The possible reasons include congestion and/or hypoperfusion, leading to organ damage, impairment of function, and, ultimately, organ failure, all of which are associated with increased morbidity and mortality [2]. Interactions between HF and other organs, particularly the kidneys [3], lungs [4], and liver [5], have recently been studied. The pancreas may also be one of the affected organs, and several reports have suggested that hypoperfusion in association with HF might cause pancreatic damage [6,7,8,9,10,11]; however, chronic passive congestion in association with acute decompensated HF (ADHF) may induce atrophy of the pancreatic acinar cells and disappearance of prezymogen granules in the atrophied pancreatic acinar cells [6], which could result in decreased production or release of pancreatic exocrine enzymes. Despite this, little attention has been paid to alterations in the pancreas due to passive venous congestion associated with HF.

Malnutrition, a common problem in patients admitted to the intensive care unit, has a negative impact on clinical outcomes, such as a higher risk of infection and multiple organ dysfunction, prolonged mechanical ventilation and hospital stay, and increased morbidity and mortality [12,13]. Malnutrition is also a major consequence and clinical manifestation of pancreatic dysfunction because pancreatic enzymes are essential for the digestion of micronutrients; inadequate release of pancreatic enzymes leads to maldigestion, which results in malabsorption of fat, protein, and fat-soluble vitamins, leading ultimately to nutritional deficiencies [14,15]. Patients with HF are likely to have poor nutritional status [16,17], and coexisting poor nutritional status is associated with worse clinical outcomes in patients with stable HF and ADHF [18,19,20,21]. Our group previously published data wherein patients with ADHF had low levels of pancreatic exocrine enzymes (i.e., amylase and lipase), and the levels of pancreatic exocrine enzymes correlated directly with parameters of nutritional status and inversely correlated with indicators of cardiac congestion [22]; hence, the low levels of pancreatic exocrine enzymes [23] at the time of admission may be associated with worse clinical outcomes in patients hospitalized with ADHF. We hypothesized that low serum amylase and lipase levels would be associated with worse clinical outcomes in patients hospitalized for ADHF.

## 2. Materials and Methods

### 2.1. Study Participants

Patients admitted to the cardiac intensive care unit of Juntendo University Hospital (Tokyo, Japan) with ADHF between November 2014 and September 2016 were included in the study. ADHF was defined based on the modified Framingham criteria [24]. The exclusion criteria were as follows: patients undergoing dialysis; patients with neoplasms; patients with a known history of pancreatic disease; patients with serum amylase and lipase levels three times greater than the upper limit of normal, which is the diagnostic criterion for acute pancreatitis [25]; and patients without documented amylase and lipase levels upon admission.

The Institutional Review Board of the Juntendo University Hospital approved the study protocol (#871), which complied with the Declaration of Helsinki. Informed consent was obtained from all patients.

### 2.2. Data Collection

Baseline data were prospectively collected at the time of initial hospital admission. Serum amylase and lipase levels, as well as other baseline biochemical parameters, were measured in the early morning on the day after hospital admission. Medical history was obtained from clinical chart reviews. The estimated glomerular filtration rate (eGFR) was calculated using the Modification of Diet in Renal Disease equation with a Japanese coefficient from baseline serum creatinine levels [26]. Complete two-dimensional echocardiography was performed for each patient. Left ventricular ejection fraction (LVEF) was calculated using a modified Simpson method. The Geriatric Nutritional Risk Index (GNRI) was calculated from the serum albumin level and body mass index (BMI) obtained upon hospital admission as follows: 14.89 * (serum albumin [g/dL]) + 41.7 * (BMI/22). Because there is no established cut-off value to define low serum amylase and lipase levels, low serum amylase and lipase levels were defined as less than or equal to the first quartile of serum amylase and lipase levels, respectively. Patients were categorized into those with and without low amylase or lipase levels.

All patients were followed up at our hospital from the date of index admission until December 2016, and outcome data were obtained during a clinical visit or by reviewing the medical records for all recorded deaths. Readmission for ADHF was defined as the first unscheduled admission to the cardiology ward because of progressive symptomatic and/or hemodynamic deterioration. The adverse event as an endpoint for this study was defined as the composite of all-cause mortality or readmission because of ADHF. An independent investigator who had no role in the follow-up or treatment of patients and had no information regarding baseline parameters determined clinical events.

### 2.3. Statistical Analysis

Continuous variables were expressed as mean ± standard deviation, and categorical variables were reported as percentages. To compare the baseline characteristics among the two groups, the χ^2^ test or Fisher’s exact test for categorical variables and the *t*-test or the Mann–Whitney U-test for continuous variables were used.

The cumulative event-free survival curves were established using the Kaplan–Meier method and were compared between groups with and without low amylase and lipase levels using log-rank tests. Univariate and multivariate Cox proportional hazards regression analyses were performed to evaluate the association between baseline variables and outcomes. A univariable Cox proportional hazard analysis was carried out by considering the following as independent variables: age, sex, BMI, ischemic etiology, atrial fibrillation, history of HF, systolic and diastolic blood pressures, heart rate, LVEF, eGFR, hemoglobin, serum albumin level, GNRI, cholinesterase level, low-density lipoprotein cholesterol level, hemoglobin A1c, blood urea nitrogen (BUN) level, serum uric acid, serum sodium, serum potassium, serum C-reactive protein (CRP), serum plasma B-type natriuretic peptide (BNP), and medication use at admission. Owing to the skewed distribution, natural logarithm-transformed CRP and BNP levels were used. Variables that showed *p* < 0.10 in the univariable analyses were entered into a multivariable forward stepwise Cox proportional hazards regression model with *p* < 0.05 to enter and *p* ≥ 0.10 to remove. The BMI and serum albumin levels used in the calculation of the GNRI were excluded from the multivariate analysis. Statistical significance was set at *p* < 0.05. Statistical analyses were performed using SPSS 23.0 (IBM Corp., Armonk, NY, USA).

## 3. Results

### 3.1. Baseline Characteristics

A total of 229 patients with ADHF were admitted to our hospital between November 2014 and September 2016. Among them, 34 patients on hemodialysis or those who did not have amylase or lipase levels upon admission were excluded. In addition, 47 patients who had life-threatening malignancies and two patients who had serum amylase and lipase levels three times greater than the upper limit of normal were excluded. Finally, data from 146 patients were analyzed. Of the 146 patients, 37 (25.3%) were identified with low serum amylase (≤39 IU/L). The baseline characteristics of patients with and without low amylase levels are shown in Table 1. Patients with low serum amylase levels were younger, had a higher BMI and eGFR, lower BUN, and were less likely to have a history of HF. Furthermore, patients with lower amylase levels were less likely to use beta-blockers and diuretics than those with higher amylase levels. Serum amylase and lipase levels were significantly lower in patients with low amylase levels than in those with high amylase levels (amylase, 30.0 [9.5] IU/L versus 63.5 [29.8] IU/L, *p* < 0.001; lipase, 18.0 [10.0] IU/L vs. 31.0 [17.5] IU/L, *p* < 0.001). Nineteen patients with low amylase levels also had low lipase levels (51.4%).

On the other hand, 36 (24.7%) patients were identified as those with low serum lipase (≤17 IU/L) levels. The baseline characteristics of patients with and without low serum lipase levels are shown in Table 2. Patients with low serum lipase levels had a lower BMI and GNRI and were more likely to have an ischemic etiology than those without low serum lipase levels. Serum amylase and lipase levels were significantly lower in patients with low lipase levels than in those without (amylase, 38.0 [25.8] IU/L vs. 61.0 [32.5] IU/L, *p* < 0.001; lipase, 14.5 [4.0] IU/L vs. 31.0 [15.5] IU/L, *p* < 0.001). Eighteen (50.0%) patients with low lipase levels also had low amylase levels.

### 3.2. Outcomes

**Figure 1:**
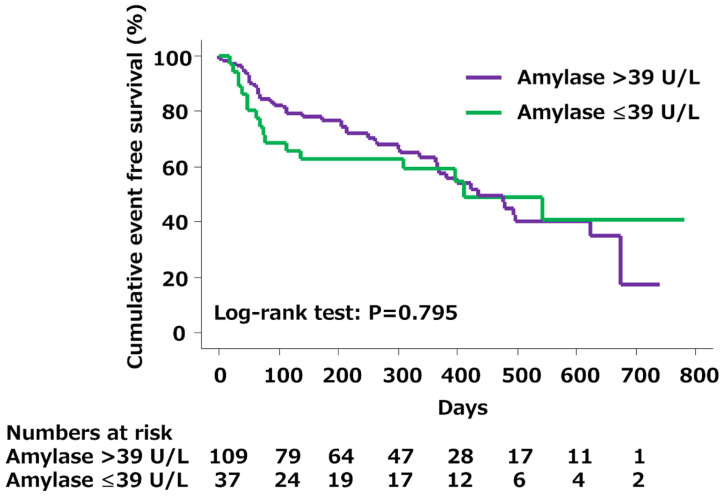
Kaplan–Meier survival curves in patients with and without low amylase. There was no significant difference in the cumulative event-free survival between patients with and without low amylase levels (≤39 U/L; log-rank test, *p* = 0.795).

**Figure 2:**
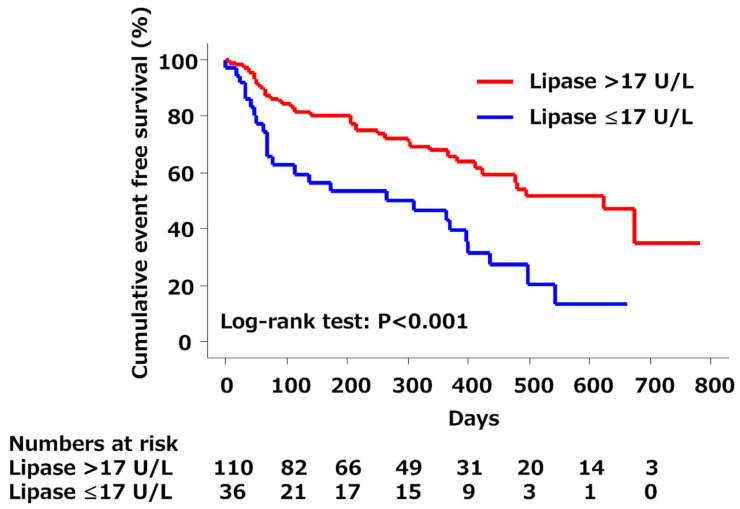
Kaplan–Meier survival curves in patients with and without low lipase. There was a significantly worse cumulative event-free survival in patients with low lipase levels (≤17 U/L) than in those with high lipase levels (log-rank test, *p* < 0.001).

All patients were followed in our institution, and all clinical events were collected. With a median follow-up of 241 days (interquartile range, 337 days), 63 clinical events occurred (43.2%). In 17 (45.9%) patients with low amylase levels, six died (16.2%) and 11 were re-hospitalized (29.7%). There was no significant difference in the cumulative event-free survival curves between patients with and without low amylase levels (Figure 1). On the other hand, in 25 (69.4%) patients with low lipase levels, 10 died (27.8%) and 15 were re-hospitalized (41.7%). The cumulative event-free survival curves differed significantly between the two groups (Figure 2).

In the univariate analysis, patients with low amylase levels had no significantly greater risk of clinical events compared to those with high amylase levels (hazard ratio [HR]: 1.08; 95% confidence interval [CI]: 0.62–1.88; *p* = 0.796; Table 3); thus, low serum amylase levels were not entered into the multivariate analysis. On the other hand, patients with low lipase levels had a significantly increased risk of clinical events compared to those with high lipase levels (HR: 2.45; 95% CI: 1.47–4.08; *p* = 0.001; Table 3). Other variables that showed *p* < 0.1 in the univariate analyses are summarized in Table 3. In the multivariate analysis, low lipase level was associated with long-term worse outcomes (HR: 1.96; 95% CI: 1.16–3.32; *p* = 0.012) in addition to age, history of HF, cholinesterase level, and HbA1c.

## 4. Discussion

We evaluated the prognostic value of serum pancreatic exocrine enzyme levels in patients with ADHF and found several novel insights into the pathophysiology of HF with respect to damage to other organs, such as the pancreas. First, although there was no significant difference in the event-free survival between patients with and without low amylase levels, the cumulative event-free survival curve was significantly worse for patients with low lipase levels than for those with high lipase levels. Second, in the multivariate analysis, a low lipase level was associated with worse long-term outcomes. These findings suggest that in patients with ADHF, although low amylase levels on admission may not be an independent predictor, low lipase levels on admission can be an independent predictor of all-cause mortality and/or rehospitalization for ADHF.

A previous study has investigated the diagnostic utility of serum amylase and lipase levels, which generally leads to irreversible dysfunction of the exocrine pancreatic function, and the correlation between severe exocrine insufficiency and low pancreatic enzyme levels is well-known in chronic pancreatitis [27]. Because pancreatic exocrine enzymes are essential for the digestion of nutrients, the decreased release of pancreatic exocrine enzymes leads to maldigestion, resulting in the malabsorption of fat, protein, and fat-soluble vitamins that ultimately contribute to poor nutritional status [14]. Indeed, in the present study, compared with ADHF patients without low serum lipase levels, those with low serum lipase levels had lower GNRI, indicating poor nutritional status. Poor nutritional status is generally associated with worse clinical outcomes in patients with ADHF [20,21]. Interestingly, in the present study, although GNRI was associated with poor clinical outcomes in the univariate analysis, multivariate analysis indicated that low serum lipase levels, rather than GNRI, were independently associated with poor clinical outcomes.

Low serum pancreatic enzyme levels in patients with ADHF may be associated with functional and/or organic damage to pancreatic tissues. Organ injury and impairment are commonly observed in patients with ADHF, and hypoperfusion and/or congestion are the essential pathophysiological mechanisms of impaired organ function [2]. It was shown that ischemia in association with hypoperfusion was an important risk factor for the development of pancreatic injury [6,7,10,11]. An acute increase in serum pancreatic enzyme levels after exposure of the pancreas to ischemia/hypoperfusion, even for a brief duration, has been reported. Moreover, the percentage increase in serum levels of pancreatic amylase and lipase over basal values, an indicator of the degree of acinar cell injury, was significantly greater in patients with longer clamping times [7]; thus, pancreatic ischemia/hypoperfusion results in increased serum pancreatic enzyme levels. In contrast, in the pancreas, Vonglahn et al. found that chronic passive congestion due to ADHF may lead to acinar cell atrophy and the disappearance of prezymogen granules [6], both of which may result in decreased serum pancreatic exocrine enzyme levels. Despite the lack of effect of the interval from the first episode of ADHF to death, a longer duration of congestion related to the recent ADHF episode was associated with more advanced changes in pancreatic tissue [6]. Therefore, low lipase levels in the present study may be due to predisposing congestion in association with chronic HF before an ADHF episode. This suggests that congestion in association with ADHF can alter pancreatic tissue morphology and possibly exocrine function and, in turn, influence the nutritional status and long-term outcomes.

In the present study, the cumulative event-free survival curves were significantly worse in patients with low lipase levels but not in patients with low amylase levels. There are two reasons for this lack of association between low amylase levels and clinical outcomes. First, amylase is ordinarily found in several other organs, such as the salivary glands, liver, biliary tree, duodenum, stomach, esophagus, lung, heart, and fallopian tubes; and second, amylase can be ectopically produced by a number of solid organs and hematological malignancies [28,29]. Second, amylase is known to occur in two major isoenzymatic forms, the pancreatic type (p-amylase) and salivary type (s-amylase), which represent approximately 40% and 60% of the total amylase activity, respectively. These isoforms differ primarily in terms of their net electrical charge, which allows their separation through techniques such as electrophoresis, isoelectric focusing, and chromatography [30,31]. For these reasons, serum lipase levels, rather than serum amylase levels, may be associated with patient outcomes.

Our study has some limitations. First, it was conducted in a single academic center and included only Japanese patients. Our findings need to be examined in a larger population to increase the generalizability. Second, we cannot exclude the possibility that unmeasured factors may have influenced some of our findings, even after considering confounding factors. For instance, in our hypothesis, the duration of HF may affect serum levels of pancreatic exocrine enzymes, as well as clinical outcomes. However, we do not have such information in the present study. In addition, dietary fat may be associated with serum lipase levels. This is more likely in patients with HF, who are recommended a low-fat diet, and in those with malnutrition, who have a potentially insufficient dietary fat intake, affecting lipase levels. Third, a recent study suggested that in patients with acute heart failure requiring intravenous loop diuretics, serum amylase and lipase levels increased from the baseline before the start of treatment until the 24 h following admission [32]. Baseline parameters, including serum amylase and lipase levels, were measured in the early morning on the next day of hospital admission. Thus, baseline measurements were conducted within the first 12 h in most cases. Nevertheless, in some cases, serum amylase and lipase were already beginning to increase; thus, serial measurements to clarify whether low levels of lipase are associated with long-term clinical outcomes are warranted. Finally, we did not directly assess the pancreatic exocrine function; therefore, investigations wherein specific pancreatic exocrine function assessments are performed, such as pancreatic function diagnostic tests or fecal elastase-1 determination, are required. Further mechanistic studies are required, such as a direct assessment of exocrine pancreatic function and those that address whether exocrine pancreatic dysfunction is associated with malnutrition and/or poor clinical outcomes.

## 5. Conclusions

Based on the findings of the present study, low serum lipase levels on admission may be a predictor of long-term clinical outcomes in patients with ADHF.

## Figures and Tables

**Table 1:** Baseline patient characteristics according to amylase level.

Variables	All *n* = 146	Amylase ≤ 39 IU/L *n* = 37	Amylase > 39 IU/L *n* = 109	*p*
Age, years	71.5 ± 14.3	66.6 ± 16.4	73.1 ± 13.1	0.027
Female gender, *n* (%)	50 (34.2)	11 (29.7)	39 (35.8)	0.639
Body mass index, kg/m^2^	23.6 ± 5.0	25.3 ± 6.4	23.0 ± 4.3	0.045
Ischemic etiology, *n* (%)	64 (43.8)	13 (35.1)	51 (46.8)	0.297
AF, *n* (%)	74 (50.7)	16 (43.2)	58 (53.2)	0.391
History of heart failure, *n* (%)	75 (51.4)	13 (35.1)	62 (56.9)	0.036
Systolic blood pressure, mmHg	135.9 ± 29.8	141.6 ± 29.8	133.9 ± 29.8	0.219
Diastolic blood pressure, mmHg	79.3 ± 19.3	84.1 ± 18.9	77.7 ± 19.3	0.088
Heart rate, per min	91.5 ± 24.6	95.0 ± 24.2	89.7 ± 24.3	0.329
Left ventricular ejection fraction, %	42.1 ± 17.0	38.4 ± 15.1	43.1 ± 17.6	0.119
eGFR, mL/min/1.73 m^2^	53.8 ± 25.1	67.8 ± 22.3	49.0 ± 24.2	<0.001
Hemoglobin, g/dL	12.0 ± 2.3	12.5 ± 2.3	11.8 ± 2.3	0.141
Albumin, g/dL	3.1 ± 0.5	3.1 ± 0.5	3.1 ± 0.5	0.491
GNRI	91.0 ± 12.3	93.5 ± 15.4	90.0 ± 10.8	0.202
Cholinesterase, U/L	206.3 ± 64.5	211.7 ± 65.7.0	204.7 ± 64.5	0.625
LDL cholesterol, mg/dL	98.4 ± 31.7	101.1 ± 34.0	96.5 ± 31.4	0.545
HbA1c, %	6.3 ± 1.2	6.5 ± 1.4	6.3 ± 1.1	0.346
BUN, mg/dL	26.1 ± 16.1	19.8 ± 11.4	28.3 ± 17.0	0.001
Uric acid, mg/dL	7.2 ± 2.4	7.9 ± 2.5	7.0 ± 2.3	0.067
Sodium, mmol/L	139.5 ± 3.8	139.8 ± 3.8	139.4 ± 3.8	0.585
Potassium, mmol/L	4.0 ± 0.6	4.1 ± 0.7	4.0 ± 0.6	0.744
CRP, mg/dL	0.9 (3.0)	1.0 (2.7)	3.1 (3.1)	0.688
BNP, pg/mL	811.0 (953.5)	822.0 (1064.1)	811.0 (957.5)	0.577
Beta blockers, *n* (%)	68 (46.6)	9 (24.3)	59 (54.1)	0.003
ACE-Is/ARBs, *n* (%)	74 (50.7)	13 (35.1)	61 (56.0)	0.046
MR antagonists, *n* (%)	34 (16.4)	4 (10.8)	20 (18.3)	0.417
Diuretics, *n* (%)	76 (52.1)	13 (35.1)	63 (57.8)	0.028

Continuous variables are expressed as mean ± standard deviation or median (interquartile range), while categorical variables are expressed as numbers (%). ACE-I, angiotensin-converting enzyme inhibitor; AF, atrial fibrillation; ARB, angiotensin II receptor blocker; BNP, B-type natriuretic peptide; BUN, blood urea nitrogen; CRP, C-reactive protein; eGFR, estimated glomerular filtration rate; GNRI, Geriatric Nutritional Risk Index; HbA1c, glycosylated hemoglobin A1c; LDL, low-density lipoprotein; MR, mineral corticoid receptor.

**Table 2:** Baseline patient characteristics according to lipase level.

Variables	All *n* = 146	Lipase ≤ 17 IU/L *n* = 36	Lipase > 17 IU/L *n* = 110	*p*
Age, years	71.5 ± 14.3	73.6 ± 14.8	70.7 ± 14.1	0.301
Female gender, *n* (%)	50 (34.2)	15 (41.7)	35 (31.8)	0.380
BMI, kg/m^2^	23.6 ± 5.0	22.2 ± 3.9	24.0 ± 5.3	0.049
Ischemic etiology, *n* (%)	64 (43.8)	22 (61.1)	42 (38.2)	0.027
AF, *n* (%)	74 (50.7)	14 (38.9)	60 (54.4)	0.150
History of heart failure, *n* (%)	75 (51.4)	18 (50.0)	57 (51.8)	0.999
Systolic blood pressure, mmHg	135.9 ± 29.8	132.6 ± 32.6	137.0 ± 29.1	0.499
Diastolic blood pressure, mmHg	79.3 ± 19.3	75.5 ± 18.1	80.7 ± 19.6	0.166
Heart rate, per min	91.5 ± 24.6	87.4 ± 22.1	92.3 ± 24.9	0.293
Left ventricular ejection fraction, %	42.1 ± 17.0	41.8 ± 15.6	42.2 ± 17.6	0.887
eGFR, mL/min/1.73 m^2^	53.8 ± 25.1	59.2 ± 25.2	52.3 ± 24.9	0.051
Hemoglobin, g/dL	12.0 ± 2.3	11.5 ± 2.0	12.2 ± 2.4	0.109
Albumin, g/dL	3.1 ± 0.5	3.0 ± 0.5	3.2 ± 0.4	0.202
GNRI	91.0 ± 12.3	85.9 ± 8.8	92.5 ± 12.7	0.001
Cholinesterase, U/L	206.3 ± 64.5	190.2 ± 50.6	209.9 ± 68.5	0.150
LDL cholesterol, mg/dL	98.4 ± 31.7	89.6 ± 25.8	100.7 ± 33.5	0.133
HbA1c, %	6.3 ± 1.2	6.5 ± 1.0	6.3 ± 1.2	0.293
BUN, mg/dL	26.1 ± 16.1	25.9 ± 13.5	26.1 ± 16.9	0.928
Uric acid, mg/dL	7.2 ± 2.4	7.0 ± 2.6	7.4 ± 2.3	0.342
Sodium, mmol/L	139.5 ± 3.8	139.4 ± 3.8	139.5 ± 3.8	0.691
Potassium, mmol/L	4.0 ± 0.6	4.1 ± 0.7	4.0 ± 0.6	0.759
CRP, mg/dL	0.9 (3.0)	2.0 (5.3)	0.7 (2.4)	0.062
BNP, pg/mL	811.0 (953.5)	928.0 (1280.3)	806.0 (869.0)	0.213
Beta blockers, *n* (%)	68 (46.6)	17 (47.2)	51 (46.4)	0.999
ACE-Is/ARBs, *n* (%)	74 (50.7)	17 (47.2)	57 (51.8)	0.774
MR antagonists, *n* (%)	34 (16.4)	3 (8.3)	21 (19.1)	0.210
Diuretics, *n* (%)	76 (52.1)	17 (47.2)	59 (53.6)	0.634

Continuous variables are expressed as mean ± standard deviation or median (interquartile range), while categorical variables are expressed as numbers (%). ACE-I, angiotensin-converting enzyme inhibitor; AF, atrial fibrillation; ARB, angiotensin II receptor blocker; BNP, B-type natriuretic peptide; BUN, blood urea nitrogen; CRP, C-reactive protein; eGFR, estimated glomerular filtration rate; GNRI, geriatric nutritional risk index; HbA1c, glycosylated hemoglobin A1c; LDL, low-density lipoprotein; MR, mineral corticoid receptor.

**Table 3:** Results of the univariate and multivariate Cox proportional hazard regression.

	Univariate			Multivariate		
	HR	95% CI	*p*	HR	95% CI	*p*
Age (1 year increase)	1.03	1.01–1.05	0.015	1.03	1.00–1.05	0.016
History of heart failure (yes)	2.43	1.43–4.14	0.001	2.70	1.55–4.72	0.001
Ischemic etiology (yes)	2.19	1.32–3.62	0.002	-	-	-
Diastolic blood pressure (1 mmHg increase)	0.98	0.97–0.99	0.016	-	-	-
Hemoglobin (1 g/dL increase)	0.86	0.77–0.95	0.005	-	-	-
Albumin (1 g/dL increase)	0.42	0.24–0.74	0.003	-	-	-
Cholinesterase (1 U/L increase)	0.99	0.98–0.99	<0.001	0.99	0.98–0.99	0.001
LDL cholesterol (1 mg/dL increase)	0.98	0.97–0.99	0.002	-	-	-
HbA1c (1% increase)	1.23	1.02–1.49	0.032	1.43	1.16–1.76	0.001
BUN (1 mg/dL increase)	1.03	1.01–1.04	<0.001	-	-	-
eGFR (1 mL/min/1.73 m increase)	0.99	0.98–0.99	0.013	-	-	-
GNRI (1 increase) *	0.97	0.95–0.99	0.013	-	-	-
Sodium (1 mmol/L increase)	0.92	0.87–0.98	0.011	-	-	-
Log-CRP (1 increase)	1.18	0.99–1.41	0.061	-	-	-
Log-BNP (1 increase)	1.44	1.04–2.00	0.028	-	-	-
Use of Beta blocker (yes)	1.99	1.20–3.31	0.008	-	-	-
Use of MR antagonists (yes)	1.72	0.91–3.24	0.094	-	-	-
Use of diuretics (yes)	2.00	1.19–3.35	0.009	-	-	-
Low amylase (yes)	1.08	0.62–1.88	0.796	-	-	-
Low lipase (yes)	2.45	1.47–4.08	0.001	1.96	1.16–3.32	0.012

* Albumin level was not included in the multivariate analysis because it was already used to calculate the GNRI. BNP, brain natriuretic peptide; BUN, blood urea nitrogen; CI, confidence interval; CRP, C-reactive protein; eGFR, estimated glomerular filtration rate; GNRI, geriatric nutritional risk index; HbA1c, glycosylated hemoglobin A1c; HR, hazard ratio; LDL, low-density lipoprotein; MR, mineral corticoid receptor.

## Data Availability

The datasets generated and analyzed in the current study are not publicly available but are available from the corresponding author upon reasonable request.

## Abbreviations

The following abbreviations are used in this manuscript:

HF	Heart failure
ADHF	Acute decompensated heart failure
eGFR	Estimated glomerular filtration rate
LVEF	Left ventricular ejection fraction
GNRI	Geriatric Nutritional Risk Index
BMI	Body mass index
BUN	Blood urea nitrogen
CRP	C-reactive protein
BNP	B-type natriuretic peptide
HR	Hazard ratio
CI	Confidence interval
